# Relationship between Revised Graduated Index (R-GINDEX) of Prenatal Care Utilization & Preterm Labor and Low Birth Weight

**DOI:** 10.5539/gjhs.v6n3p131

**Published:** 2014-02-27

**Authors:** Tahereh Tayebi, Zeinab Hamzehgardeshi, Marjan Ahmad Shirvani, Marjaneh Dayhimi, Mahmonir Danesh

**Affiliations:** 1 Department of Midwifery, Mazandaran University of Medical Sciences, Sari, Islamic Republic of Iran; 2 Traditional and Complementary Medicine Research Centre, Mazandaran University of Medical Sciences, Sari, Islamic Republic of Iran; 3 Department of Midwifery and Reproductive Health, Mazandaran University of Medical Sciences, Sari, Islamic Republic of Iran; 4 Department of Midwifery, International Branch of Shahid Beheshti Medical University, Tehran, Iran

**Keywords:** prenatal care, revised G Index, premature labor, low birth weight, neonate

## Abstract

Prenatal care refers to accurate and consistent performance of the principles important to maintain healthy pregnancy outcomes and also for mother and child health. One of the new indices to assess the adequacy of care is Revised Graduated Index of Prenatal Care Utilization (R-GINDEX). The study aims to assess the relationship between quantitative prenatal care factors and preterm labor and low birth weight using R-GINDEX. This historical cohort study has been conducted on 420 mothers during the first two years after delivery in 2010. The adequacy of care was calculated by R-GINDEX. Based on this index, participants have been divided into three care groups including inadequate, adequate and intensive care groups. A significant relationship has been found between R-GINDEX and preterm birth and low birth weight (P<0.05). Thus the probability of premature labor in inadequate care group (RR=3.93) and low birth weight (RR= 2.53) was higher than that of the adequate and intensive care group. The results showed that the quantity of prenatal care is effective in reducing preterm birth and low birth weight.

## 1. Introduction

According to the most recent estimates, 343,000 mothers died in 2008 due to complications related to pregnancy and childbirth (Hogan et al.,2010). Many cases of maternal and fetal mortalities and morbidities as well as stillbirth, preterm birth and low birth weight are because of inadequate and inappropriate prenatal care. Appropriate prenatal care can greatly reduce most of the maternal and child complications and problems in future. The overall rate of fetal death was 2.7 per 1000 births in women who received prenatal care versus 14.1 per 1000 in women not receiving any prenatal care. Researchers reported that lack of prenatal care is associated with 3.3 times increase in the relative risk of stillbirth and two times rise in the risk of preterm labor (Cunningham, Gant, Leveno, & Larry, 2010).

More recently, a research reviewed a 10 year retrospective study where the risk of preterm birth among the adolescents who received inadequate prenatal care has been assessed. They found that the women with no prenatal care had nearly 8-fold higher risk of preterm birth (odds ratio [OR], 7.9; 95% confidence interval [CI], 6.1 - 10.3) compared with those who attended 75% - 100% of the recommended visits (Debiec, Paul, Mitchell, & Hitti, 2010).

Adequate prenatal care provides an opportunity for consultation and reduces complications related to pregnancy and delivery (Miranda et al.,2010). In a study with the aim to determine the factors associated with inadequate prenatal care in Ecuadorian women, it has been discovered that the inadequate care was in 75.5% of cases (by Kessner index) while an adverse outcome of the prior pregnancy (abortion, intrauterine fetal demise, or ectopic pregnancy) increased this risk (Paredes, Hidalgo, Chedraui, Palma, & Eugenio, 2005). Researchers have demonstrated that adequate prenatal care was an effective intervention to improve pregnancy outcomes (Stringer, 1998). On the other hand, a group of researchers demonstrated that reducing the number of visits had no harmful effects on maternal and neonatal outcomes in low-risk pregnant women (Walker, McCully, & Vest, 2001). In another study performed by the Michigan College of Nursing to assess satisfaction and adequacy of prenatal care among low-income rural women, 50% of women with less adequate care were satisfied with the care and optimal outcomes of pregnancy. There was no difference between this group and the group of women who received adequate care (Omar & Schiffman, 2000).

A new index used to provide more accurate and comprehensive measurements of prenatal care utilization is the revised G Index. This index can identify the adequacy of prenatal care by the use of care starting time and care relevance number. It is worth to mention that this index does not consider the quality of service, and only measures the usefulness or adequacy of care (VanderWeele, Lantos, Siddique, & Lauderdale, 2009).

According to the previous studies derived results, there is a controversy about the effect of quantitative care factors that improve pregnancy outcomes. So that this study aims to analyze the relationship between quantitative prenatal care factors and preterm labor and low birth weight by using R-GINDEX.

## 2. Method

The samples were selected randomly out of the individuals who referred to the public health center to receive maternal-child care during the first two years after childbirth in 2010 based on inclusion and exclusion criteria. The sample size was determined according to the previous studies like the ones by Alexander and colleagues (2001), the Department of Kansas (2008) and the other researches and by statistical formulas with 95% confidence (420 women).

The review board of Shahid Beheshti University of Medical Science approved the study protocol. Inclusion criteria were 18-35 year-old mothers with singleton fetus in their last pregnancy, lack of physical and psychological illness and available family medical files. The mothers with previous history of preterm birth, low birth weight, smoking, alcohol & drug abuse and mellitus diabetes and fetal abnormalities were excluded in order to reduce confounding variables. We applied random sampling method. After receiving the information form, in case the women agreed to participate in the study, they signed the consent form.

The researcher-built questionnaire was provided including 27 questions about demographic characteristics, obstetric history, delivery data and infant features. Validity of the questionnaire was assessed by validity qualitative content method and reliability was higher than 90 by test, re- test and correlation analysis. The questionnaires were completed through daily visit and interviews with the mothers. Other information was completed from the mothers’ health file and other sources based on existing data at the health centers.

Adequacy of care was calculated by R-GINDEX. This index is one of the five indicators used to measure the adequacy of prenatal care proposed by Alexander and Kotelchuck in 1994 (newer than Kessner’s index). Three parameters associated with birth are required to calculate R-GINDEX as the following:

1-The start of cares (Trimester 1-3); 2-Gestational Age; and 3-The total number of prenatal cares.

Based on this index, participants were divided into three care groups including: inadequate, adequate and intensive care groups. Adequate care refers to the minimum recommended level of cares. Intensive care is the care much higher than the number of the recommended level and is calculated as one standard deviation more than moderate standard of cares in each period in R-GINDEX. Inadequate care includes women with both lower than the average and no prenatal care. For accurate measurement of indices, the expected number of visits was calculated according to the standardized protocol. In this protocol, eight visits have been usually done based on the gestational week for low-risk pregnancies, two visits in the first half of pregnancy(6-20weeks), and six visits in the second half (21-40weeks). Preterm birth was considered as delivery occurring before 37 weeks of gestation and low birth weight was viewed as the weight less than 2500 g at birth (Lowdermilk, Perry, & Bobak, 2004). Statistical analysis was performed using descriptive statistics, chi-square test, variance analysis, t-test, Spearman correlation coefficient and relative risk.

## 3. Results

**Figure 1:**
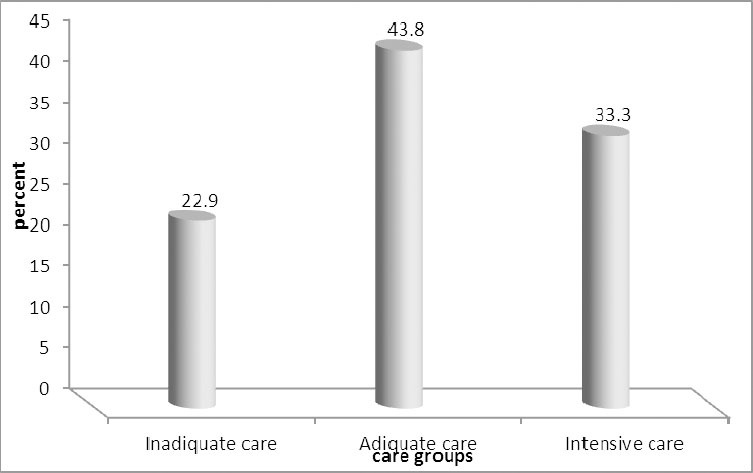
Frequency of care groups based on the R-G Index

Totally 420 women were assessed. At the first visit, most of the mothers (44.5%) were within 9-16 weeks of gestation. The majority of mothers (51.9%) received 5-8 times of care. Adequate care was the most common category of maternal care (Figure 1).

There was significant difference in the level of education, the type of delivery, the first visit, the total number of cares, gestational age, birth weight, complete prenatal tests, participation in child birth preparation classes and the number of ultrasonography among the care groups (Tables 1 and 2).

**Table 1:** The demographic characteristics and frequency of care groups based on them

Demographic characteristics	Total sample	Adequacy of care (M±SD)			P value
		Inadequate	Adequate	Intensive	
Age	25.5±4.3	26.12± 4.77	26.47± 4.27	26.82± 4.24	NS^†^
BMI^‡^ (M±SD)	24.82±3.58	25.40± 4.09	24.65± 3.44	24.66± 3.37	NS
Job	N(%)	N(%)	N(%)	N(%)	
House wife	363(86.4)	82(22.6)	155(42.7)	126(34.7)	NS
Employer	57(13.6)	14(24.6)	29(50.9)	14(24.6)	
Education					
Primary	12(2.9)	1(1.0)	5(2.7)	6(4.3)	
High school	63(15.0)	8(8.3)	29(15.8)	26(18.6)	0.04
Diploma	229(54.5)	49(51.0)	105(57.1)	75(53.6)	
University	116(27.6)	38(39.6)	45(24.5)	33(23.6)	

† Non significant

‡ Body Mass Index.

**Table 2:** The obstetrics characteristics and frequency of care groups based on them

Obstetrics characteristics	Total sample	Adequacy of care (M±SD)			P value
		Inadequate	Adequate	Intensive	
First visit (week)	14.48±6.84	21.40± 6.96	14.20± 5.64	10.10± 3.71	0.000
Frequency of cares	4.80±1.91	2.34± 0.75	4.48± 0.50	6.89± 1.27	0.000
Gestational age(Week)	38.40±1.39	37.78± 1.40	38.25± 1.23	39.03± 1.33	0.000
Neonatal weight(Gr)	3203.31±469.56	3117.6± 505.95	3174.08± 450.65	3300.5± 454.52	0.007
Ultrasonography	2.84±1.28	2.60±1.34	2.82±1.24	3.01±1.28	0.05
Complete tests N(%)					0.000
Yes	409(97.4)	87(21.3)	182(44.5)	140(34.2)	
No	11(2.6)	9(81.8)	2(18.2)	0	
Participation in childbirth classes				0.000
Yes	135(32.1)	17(12.6)	59(43.7)	59(43.7)	
No	285(67.9)	79(27.7)	125(43.9)	81(28.4)	
Delivery type					0.003
Vaginal	109(26)	13(13.5)	50(27.2)	46(32.9)	
Cesarean	311(74)	83(86.5)	134(72.8)	94(67.1)	

Overall, 26 mothers (6.2%) had preterm delivery. The maximum rate of preterm birth was seen in the inadequate care and the minimum in intensive care group. There was a significant correlation between R-G Index and preterm delivery (Table 3), as the relative risk (RR) of preterm delivery in the inadequate care group was 3.93 times more than that of the adequate and intensive care groups.

**Table 3:** The relation of preterm labor with R-G Index and its components

R-G Index and its components	Preterm	labor	P value
Index category	Yes N(%)	No N(%)	0.000
Inadequate	14(14.6)	82(85.4)	
Adequate	8(4.3)	176(95.7)	
Intensive	4(2.9)	136(97.1)	
	M±SD	M±SD	
Onset of care (week)	17.19 ±8.57	14.30 ± 6.69	NS
Frequency of cares	3.62 ± 2.65	4.88± 1.83	0.001

Also, 35 women (8.3%) had low birth weight that was the highest in inadequate care and the lowest in intensive care group. A significant correlation was observed between low birth weight and care-given groups (Table 4). Thus low birth weight among the mothers with inadequate maternal care was higher than that in women who received adequate care or intensive care (RR= 2.53).

**Table 4:** The relation of low birth weight with R-G Index and its components

R-G Index and its components	Low birth	weight	P value
Index category	Yes N(%)	No N(%)	0.000
Inadequate	15(15.6)	81(84.4)	
Adequate	14 (7.6)	170(92.4)	
Intensive	6(4.3)	134(95.7)	
	M±SD	M±SD	
Onset of care (week)	8.17 ± 17.17	6.67 ± 14.23	0.04
Frequency of cares	2.5±4.0	1.8 3± 4.87	0.01

The mean number of cares was lower in preterm birth and low birth weight (Table 3 and 4). Most of the mothers (93.8%) were taking ferrous pills daily, but no significant correlation was found between ferrous intake and preterm birth (P=0.47) and also low birth weight (P= 0.53). While there was a significant correlation between multivitamin- folic acid intake & preterm birth (p=0.02 & 0.004, respectively) and low birth weight (p=0.03 & P=0.004, respectively).

## 4. Discussion

The results of this study showed that the highest percent of the mothers were in the adequate care group. In the similar studies based on the recommended index by America Academy of Obstetrics and Gynecology (ACOG), most of the women were in the intensive and adequate care groups (Alexander & Kotelchuck, 2001; Heaman, Newburn-Cook, Green, Elliott, & Helewa, 2008). The main finding in this study was an increased risk of preterm birth with inadequate care in low-risk pregnant women so that the relative risk of preterm birth in inadequate care group was 3.93 times higher than that of the mothers in the other groups. Similarly, another study (Paredes et al.,2005) suggested that the rate of preterm birth was 7.2% in inadequate care that was approximately twice than the rate of the adequate care group (3.5%). Using R-GINDEX, insufficient care was significantly correlated with preterm labor (OR=1.2). In other words, the rate of preterm birth increased by 20% via inadequate care. Another research showed that the risk of preterm birth in the women with inadequate care was 2 times than that of the women receiving adequate or average care (OR= 2.1) (Krueger & Scholl, 2000). The study results also revealed that the group prenatal care results in higher birth weight, especially “in the children who had been born preterm (Ickovics et al.,2007). American’s Department of Health and Human Services study demonstrated that in the mothers who had no prenatal care, the risk of low birth weight and infant mortality risk increased 3 times and 5-times, respectively.

Another result of this study was a meaningful relationship found between mothers’ education level and providing care to the mothers. So, the higher educated mothers (high school or college level) received more adequate care than the other groups. There was a significant relationship between the participation of the mothers in childbirth preparation classes and performing adequate care (P<0.05). Also, using R-GIndex, the probabilities of low birth weight are higher in low-risk women with inadequate prenatal care, thus the risk is 2.53 times more than that of the other groups. These results suggest that through mass media, the development of maternal caring programs, mothers referring to health centers and participation in childbirth preparation classes can reduce the role of low level of education on the rate of adverse outcomes such as low birth weight and preterm birth. A similar study showed that there is a correlation between inadequate care and low birth weight based on R-GINDEX (OR=1.1). According to the survey results and comparison with similar studies, again the effect of prenatal care quantity on pregnancy outcome, especially preterm birth and low birth weight can be emphasized.

In our study, the existence of the specific criteria such as the previous history of preterm delivery and low birth weight or chronic disease as the factors influencing pregnancy outcome were excluded. Thus according to the previous study in Iran and considering the effect of the mentioned variables on pregnancy outcomes, it can be expected that the rate of premature birth and low birth weight in high-risk pregnant women is much more than that of the results of present study (Vahdaninia, Tavafian, & Montazeri, 2008).

The limitations of this study were the existence of some important maternal factors such as socioeconomic condition, race, ethnicity and health behaviors that were not controlled and can affect the care patterns and pregnancy outcomes. Further studies are suggested to survey the relationship between the quantity of prenatal care and the other adverse pregnancy outcomes. In the recent years, some countries such as Iran reduced the total number of prenatal cares with the aim to increase the quality of cares, while this research concluded that many services need to be provided in a timely manner, so increasing the interval of cares may result in losing many opportunities for the management of gradual or sudden complications in pregnancy.

## 5. Conclusion

The results showed that the quantity of prenatal care is effective in reducing preterm birth and low birth weight.

## References

[ref1] Alexander G. R, Kotelchuck M (2001). Assessing the role and effectiveness of prenatal care: history, challenges, and directions for future research. Public Health Reports.

[ref2] Cunningham F. G, Gant N. F, Leveno K. J, Larry C (2010). Williams obstetrics.

[ref3] Debiec K. E, Paul K. J, Mitchell C. M, Hitti J. E (2010). Inadequate prenatal care and risk of preterm delivery among adolescents: a retrospective study over 10 years. American journal of obstetrics and gynecology.

[ref4] Heaman M. I, Newburn-Cook C. V, Green C. G, Elliott L. J, Helewa M. E (2008). Inadequate prenatal care and its association with adverse pregnancy outcomes: a comparison of indices. BMC Pregnancy and Childbirth.

[ref5] Hogan M. C, Foreman K. J, Naghavi M, Ahn S. Y, Wang M, Makela S. M, Murray C. J (2010). Maternal mortality for 181 countries, 1980-2008: a systematic analysis of progress towards Millennium Development Goal 5. The Lancet.

[ref6] Ickovics J. R, Kershaw T. S, Westdahl C, Magriples U, Massey Z, Reynolds H, Rising S. S (2007). Group prenatal care and perinatal outcomes: a randomized controlled trial. Obstetrics and gynecology.

[ref7] Krueger P. M, Scholl T. O (2000). Adequacy of prenatal care and pregnancy outcome. JAOA: Journal of the American Osteopathic Association.

[ref8] Lowdermilk D. L, Perry S. E, Bobak I. M (2004). Maternity & women’s health care.

[ref9] Miranda A. E, Trindade C. R, Nunes R. H, Marba E. F, Fernandes M. C, Quarto G. H. A, França L. C (2010). Factors associated with prenatal care and seeking assistance in public hospitals in Vitoria, Espirito Santo, Brazil. Women & Health.

[ref10] Omar M, Schiffman R (2000). Satisfaction and adequacy of prenatal care utilization among rural low-income women. Outcomes management for nursing practice.

[ref11] Paredes I, Hidalgo L, Chedraui P, Palma J, Eugenio J (2005). Factors associated with inadequate prenatal care in Ecuadorian women. International Journal of Gynecology & Obstetrics.

[ref12] Stringer M (1998). Issues in determining and measuring adequacy of prenatal care. Journal of perinatology: official journal of the California Perinatal Association.

[ref13] Vahdaninia M, Tavafian S. S, Montazeri A (2008). Correlates of low birth weight in term pregnancies: a retrospective study from Iran. BMC Pregnancy and Childbirth.

[ref14] VanderWeele T. J, Lantos J. D, Siddique J, Lauderdale D. S (2009). A comparison of four prenatal care indices in birth outcome models: comparable results for predicting small-for-gestational-age outcome but different results for preterm birth or infant mortality. Journal of clinical epidemiology.

[ref15] Walker D. S, McCully L, Vest V (2001). Evidence-Based Prenatal Care Visits: When Less Is More. Journal of Midwifery & Women’s Health.

